# Environmental DNA Metabarcoding Reveals Diversity and Spatiotemporal Variations of Benthic Invertebrates in Hun River Basin, China

**DOI:** 10.3390/biology15181597

**Published:** 2026-09-10

**Authors:** Peng Hou, Xin Ma, Li Liu, Ying Men, Jianfeng Wang, Ding Yang, Chuntian Zhang

**Affiliations:** 1 College of Life Science and Bioengineering, Shenyang University, Shenyang 110044, China; hp_0829@syu.edu.cn (P.H.); maxin20001105@163.com (X.M.); liujiali0229@163.com (L.L.); menying1105@163.com (Y.M.); wangjf80@126.com (J.W.); 2 Department of Entomology, College of Plant Protection, China Agricultural University, Beijing 110193, China; dyangcau@126.com; 3 College of Life Science, Shenyang Normal University, Shenyang 110034, China

**Keywords:** biodiversity monitoring, eDNA metabarcoding technique, benthic invertebrates, spatiotemporal patterns, freshwater ecosystems

## Abstract

Freshwater biodiversity underpins ecosystem functioning and human well-being. As the most diverse taxonomic groups in freshwater ecosystems, benthic invertebrates are widely employed as bioindicators for aquatic monitoring and health assessment. In this study, environmental DNA (eDNA) metabarcoding was used to reveal the diversity and spatiotemporal variations in benthic invertebrates in the Hun River Basin (the middle and upper reaches). Sampling was conducted in spring, summer, and autumn, with sites arranged to cover both anthropogenic activities and watershed environmental conditions. A total of 3 phyla, 7 classes, 18 orders, 56 families, 114 genera, and 161 species were detected. Insecta was the predominant class, and the combined EPT taxa (Ephemeroptera, Plecoptera, and Trichoptera) along with Diptera accounted for 64.71%, 74.18%, and 68.75% of the total taxa in spring, summer, and autumn. Community composition exhibited variations across both seasonal and spatial scales, as reflected by differences in OTU richness, dominant taxa and their relative sequence abundances, and alpha diversity indices. The top 10 dominant genera were all regionally common taxa, among which seven belonged to Insecta (including four from Chironomidae), whereas *Sulcospira* was the most spatially widespread dominant genus. Alpha and beta diversity analyses revealed significant seasonal differentiation but no significant spatial structuring. The SIMPER analysis identified the key genera that contributed substantially to observed community dissimilarity among sampling groups. This study provides fundamental data for freshwater biodiversity conservation and validates the potential applicability of eDNA metabarcoding for benthic invertebrate monitoring, while also revealing the current limitations of this technique.

## Simple Summary

Environmental DNA (eDNA) metabarcoding has emerged as a revolutionary tool for freshwater ecosystem conservation and biodiversity monitoring, yet its feasibility and applicability to benthic invertebrates still require further study. In this study, eDNA metabarcoding was employed to reveal the diversity and spatiotemporal variations in benthic invertebrates in the Hun River Basin. The number of detected taxa surpassed that obtained from previous conventional survey efforts. Seasonality exerted a highly significant effect on community structure, whereas the differences among spatial groups were not significant. These findings confirm the potential applicability of eDNA metabarcoding for the spatiotemporal monitoring of benthic invertebrates in typical freshwater ecosystems, providing a valuable reference case for refining eDNA-based aquatic biomonitoring strategies.

## 1. Introduction

Anthropogenic activities and extreme climatic events have imposed synergistic stress on global ecosystems, driving a sustained and unprecedented loss of biodiversity [1,2,3]. Freshwater ecosystems, characterized by limited hydrological connectivity and low resilience, have been recognized as one of the most vulnerable ecosystem types worldwide, with particularly acute biodiversity erosion [4]. In contrast to other ecosystems, freshwater systems cover only approximately 0.8% of the Earth’s surface, yet they support over 10% of all known species [5]. Nevertheless, freshwater populations have plummeted by more than 80% over the past five decades [6,7]. According to the latest assessment, 7550 aquatic species are currently classified as globally threatened [8]. Freshwater biodiversity is critical to ecosystem functioning and human well-being [5,9]. Therefore, robust biodiversity monitoring, status assessment, and trend detection are necessary for defining conservation action thresholds and assessing management effectiveness.

Benthic invertebrates constitute one of the most diverse taxonomic groups in freshwater ecosystems. They not only serve as a prey base for higher trophic levels but also play critical roles in nutrient cycling, energy flow, and information transfer, thereby occupying a pivotal ecological niche [10,11,12]. Owing to their extended life cycles, wide geographical distribution, and marked sensitivity to changes in water quality, they are widely recognized as key bioindicators for monitoring freshwater ecosystems and assessing water quality status [10,13]. However, due to budgetary and technological constraints, current monitoring efforts remain insufficient [14]. Conventional benthic invertebrate monitoring techniques, which rely on morphological identification and invasive physical sampling, require considerable time and expense and damage habitats, rendering them unsuitable for monitoring over extended periods with high accuracy. In recent years, eDNA metabarcoding has emerged as a revolutionary tool in biodiversity monitoring and has been incorporated into national surveillance programs for endangered and invasive species in several countries [15,16,17,18,19]. eDNA metabarcoding is a molecular biological technique that combines high-throughput sequencing with DNA barcoding to taxonomically identify species from genetic material extracted from environmental samples (e.g., feces, secretions, sloughed epithelial cells, and gametes) [14,20]. This approach obviates the need for prior target-tissue isolation and confers substantial advantages, including noninvasiveness, high efficiency, and cost-effectiveness. It exhibits considerable potential for aquatic biodiversity monitoring and conservation [17,18,19,20,21,22]. Although this approach has been widely applied in freshwater ecosystems worldwide, its applicability and detection performance merit further investigation.

The Hun River basin (122°30′–125°15′ E, 41°00′–42°15′ N), located in east-central Liaoning Province, is a representative freshwater ecosystem in northern China. Its watershed encompasses mountainous, hilly, and plain terrains, with a mainstream length of approximately 415 km and a drainage area of roughly 11,406 km^2^ [23,24]. As a major tributary of the Liao River, the Hun River is a vital water source for industrial and domestic water supplies in Liaoning Province; consequently, its aquatic environmental quality and ecological status have attracted considerable research attention [24,25]. Historically, biodiversity surveys and monitoring of benthic invertebrates in the Hun River basin have relied primarily on conventional trawling and morphological identification [24,25,26,27,28,29,30]. Nevertheless, these traditional approaches are hindered by inherent limitations, including inadequate detection of low-abundance taxa, difficulties in precise morphological discrimination, and inaccuracies in biomass estimation [31,32]. Against this background, the present study employed eDNA metabarcoding to investigate benthic invertebrate diversity in the middle and upper reaches of the Hun River basin. The objectives were to validate the application potential of eDNA-based monitoring for benthic communities in this watershed, systematically characterize their taxonomic composition and community structure, preliminarily elucidate the spatiotemporal variations in the benthic invertebrate communities, and describe the key contributing taxa underlying the observed community dissimilarities. The results provide fundamental baseline data and a scientific basis for aquatic biodiversity conservation and ecosystem health assessment within the basin. Furthermore, this study offers a valuable regional demonstration case study for the future development and standardization of eDNA monitoring protocols for aquatic organisms.

## 2. Materials and Methods

### 2.1. Sampling Site Selection and Water Collection

**Figure 1:**
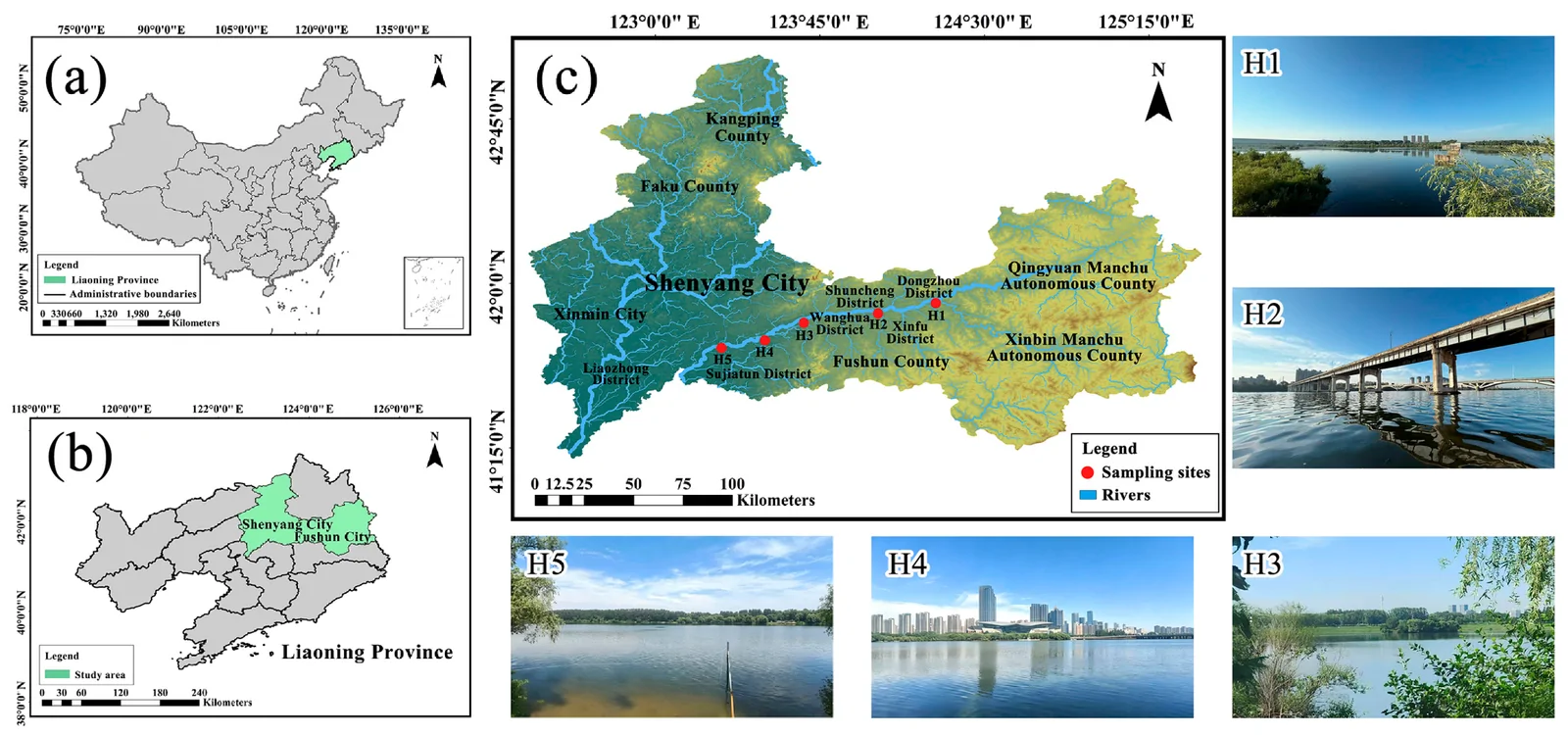
Study area location and water sampling site distribution. (**a**) Location of the study area in China. (**b**) Location of the study area in Liaoning Province. (**c**) Distribution of water sampling sites. Red dots indicate the water sampling sites, designated H1–H5 from upstream to downstream. Elevation data source: National Earth System Science Data Center (China).

The study area encompasses the middle and upper reaches of the Hun River Basin, extending downstream from the Dahuofang Reservoir in Dongzhou District (Fushun City) to the Hun River Sluice Gate in Sujiatun District (Shenyang City) (Figure 1). As a representative urban-industrial watershed, this basin constitutes a critical zone of intense anthropogenic–natural interactions, where water quality directly influences both the surrounding ecosystem and regional public livelihood [23,24]. To fully capture the seasonal variations in benthic invertebrate composition and community structure, we established five sampling sites considering local temperature, biological rhythms, and surrounding watershed conditions. Sampling was conducted in April (spring), July (summer), and October (autumn) 2025.

The sampling procedure strictly adhered to standardized protocols [33]. (1) To minimize vertical heterogeneity within the water column, water samples were sequentially collected from the surface, middle, and bottom layers in a downstream to upstream order at each site. These subsamples were mixed in equal volumes and pooled into a final 3-L composite sample. (2) To reduce interference from water flow velocity, the procedure was repeated three times at each site per season, yielding three independent replicate samples for each of the 15 season-site combinations. A total of 45 water samples were obtained (15 season-site combinations × 3 replicates). (3) A 1-L aliquot of purified water served as a negative control for each site to monitor potential exogenous DNA contamination throughout the experiment. (4) To rigorously minimize contamination risks, disposable gloves were replaced after each site, and water samples were preserved in sampling bottles pre-sterilized with 75% ethanol. (5) Immediately after collection, all samples were stored in the dark at 4 °C, transported to the laboratory, and filtered within 12 h.

### 2.2. Water Sample Processing and eDNA Extraction

The filtration apparatus and all experimental consumables were autoclaved (121 °C, 101 kPa, 20 min). Water samples were filtered using nitrocellulose membranes (0.45-μm pore size, 50-mm diameter; Delvstlab, Haining, China) on a clean bench. To prevent cross-contamination between different sampling sites, the contact surfaces of the filtration device were disinfected with bleach, rinsed three times with distilled water, and air-dried after each individual sample was processed. Separate membranes were used for replicate samples from each site. After filtration, the membranes for each water sample (three biological replicates per season-site combination) were transferred to 5 mL sterile centrifuge tubes and stored at −80 °C until DNA extraction. The membranes were cut into 2 mm fragments using sterile scissors, and genomic DNA was extracted with the Ezup Column Animal Genomic DNA Purification Kit (Sangon Biotech, Shanghai, China) according to the manufacturer’s instructions. DNA integrity was evaluated by 1% agarose gel electrophoresis, and the concentration was determined using a Qubit^®^ 4.0 fluorometer (Thermo Fisher Scientific, Waltham, MA, USA). The membranes from negative control water samples yielded no detectable DNA, and no extraneous bands were observed. All DNA samples met the quality requirements for subsequent PCR amplification.

### 2.3. PCR Amplification and High-Throughput Sequencing

The extracted DNA served as the template for PCR amplification using the universal benthic invertebrate primers mlCOIintF (5′-GGWACWGGWTGAACWGTWTAYCCYCC-3′) and dgHCO2198 (5′-TAAACTTCAGGGTGACCAAARAAYCA-3′) [34]. The PCR reaction was performed in a total volume of 50 μL, containing 2 μL of forward primer, 2 μL of reverse primer, 2 μL of template DNA, and 25 μL of 2× SanTaq PCR Master Mix, with the final volume adjusted to 50 μL using 19 μL of ddH_2_O. The thermal cycling protocol consisted of initial denaturation at 94 °C for 5 min, followed by 35 cycles at 94 °C for 30 s, annealing at 58 °C for 30 s, and extension at 72 °C for 60 s, with a final extension at 72 °C for 10 min. A negative control using ddH_2_O as the template was included to exclude potential contamination during the experimental procedures. Three replicate samples from each season–site combination were each amplified in triplicate, and the resulting amplicons from the same combination were pooled to minimize PCR bias. After validation by electrophoresis on a 2% agarose gel (with no abnormal bands detected in the PCR negative control), the 15 PCR products were submitted to Sangon Biotech (Shanghai) Co., Ltd. for sequencing library construction to facilitate subsequent analyses. The libraries were constructed via a two-round PCR strategy: the first round incorporated sample-specific barcodes to distinguish different samples, while the second round appended Illumina sequencing adapters to both ends of the amplicons. The PCR products from each round were purified using Hieff NGS™ DNA Selection Beads (Yeasen, Shanghai, China, 12601ES56). Following quality control by 2% agarose gel electrophoresis and quantification using a Qubit 3.0 fluorometer (Thermo Fisher, Waltham, MA, USA, Q32854), the qualified libraries were subjected to sequencing on the Illumina NextSeq 2000 platform (Illumina, San Diego, CA, USA).

### 2.4. Bioinformatic Analysis

The raw high-throughput sequencing reads from 15 season–site combinations were demultiplexed using cutadapt (v1.18) based on the barcode sequences. Adapter and primer sequences were trimmed, allowing up to two mismatches and a minimum overlap of 10 bp. Paired-end reads were subsequently merged using PEAR (v0.9.8) with a minimum overlap of 15 bp and a maximum mismatch ratio of 0.1. Quality control was performed using PRINSEQ (v0.20.4), retaining sequences with a quality score of Q ≥ 30 and lengths ranging from 308 to 318 bp. USEARCH (v11.0.667) was then employed for secondary quality filtering and chimera removal. Operational Taxonomic Units (OTUs) were clustered at a 97% similarity threshold, generating an OTU relative sequence abundance matrix, and the most abundant sequence within each OTU was selected as the representative sequence. Taxonomic annotation of each OTU representative sequence was conducted using BLASTn searches against the Chinese Freshwater Macroinvertebrate Barcode Database (version 2.0; https://cfmlib.njau.edu.cn), which contains extensive barcode data for local freshwater benthic fauna. This database also facilitates cross-validation with publicly available international databases, including GenBank and BOLD, thereby providing a critical reference for taxonomic assignment in this study. The alignment criteria were set as identity ≥ 97%, E-value ≤ 1 × 10^−5^, and coverage ≥ 90%. Finally, OTUs annotated as non-benthic invertebrates were excluded. Additionally, low-abundance sequences prone to causing false-positive errors (i.e., comprising < 0.005% of total reads per sample) were removed from further analyses.

### 2.5. Statistical Analysis

Alpha diversity indices, including Richness, the Shannon index, the Simpson index, and Pielou’s evenness index, were calculated to assess the diversity levels within each sample across both temporal (seasonal) and spatial dimensions. Richness is defined as the number of observed and annotated operational taxonomic units (OTUs) within a sample community. The Shannon index integrates both the richness of taxonomic units and the evenness of their relative sequence distribution, with values invariably ≥ 0. Greater taxon richness and a more equitable sequence distribution yield higher Shannon index values. The Simpson diversity index (1 − D), which ranges from 0 to 1, indicates that lower values correspond to stronger dominance of a few species and thus lower overall community diversity. Pielou’s evenness index reflects the equitability of individual distribution among taxa within a sample, with values approaching 1 suggesting a more balanced community structure. Differences in alpha diversity among samples were tested separately for seasonal and spatial effects using Kruskal–Wallis non-parametric rank-sum tests. Pairwise multiple comparisons were performed using the Dunn’s test with Bonferroni correction. For beta diversity, community dissimilarities were assessed based on the Bray–Curtis distance matrix. Non-metric Multidimensional Scaling (NMDS) was employed for ordination-based visualization of community structure. Permutational Multivariate Dispersion (PERMDISP) and Permutational Multivariate Analysis of Variance (PERMANOVA) were applied to test the significance of differences among groups, with 999 permutations under an unrestricted random permutation scheme. Pairwise post hoc comparisons were conducted using PERMANOVA, with Bonferroni correction applied to adjust for multiple comparisons. To quantify the relative contributions of temporal dynamics and spatial heterogeneity to the beta diversity of benthic invertebrate communities, average inter-group dissimilarities were separately calculated to elucidate the contribution of each factor to community pattern differentiation. Additionally, Similarity Percentage (SIMPER) analysis was performed to describe the key taxonomic groups contributing most to the observed community dissimilarities among different seasons or sites. All statistical analyses and graphical plotting were conducted using R (v4.4.0) [35] with the vegan package (v2.7-2) [36] and Origin (v2024), while the geographic information maps were generated using ArcGIS (v10.8).

## 3. Results

### 3.1. General Sequencing Data Results Based on eDNA Metabarcoding

High-throughput sequencing was performed on eDNA samples from 15 season–site combinations, generating a total of 5,670,390 raw reads. Following sequence assembly, quality filtering, chimera removal, and OTU clustering, 23,133 operational taxonomic units (OTUs) were obtained. Taxonomic annotation was conducted against the Chinese Freshwater Macroinvertebrate Barcode Database using a 97% similarity threshold, which ultimately retained 161 OTUs confidently assigned to known species. To correct for depth heterogeneity, the OTU matrix was rarefied to the minimal sample size, resulting in 48,506 high-quality reads (Table S1).

### 3.2. Taxonomic Composition of the Benthic Invertebrate Community

#### 3.2.1. Overall Taxonomic Composition

Based on eDNA metabarcoding, a total of 161 benthic invertebrate species were identified in the Hun River basin, belonging to 3 phyla, 7 classes, 18 orders, 56 families, and 114 genera (Table S2). At the phylum level, Arthropoda accounted for the largest proportion of the identified taxonomic composition, followed by Mollusca and Annelida. At the class level, Insecta was the most species-rich group, with Gastropoda and Oligochaeta as subordinate taxa. At the order level, Diptera contributed the most to the taxonomic composition, followed by Trichoptera and Ephemeroptera. Regarding families, Chironomidae represented the largest proportion among the identified families, followed by Naididae and Tabanidae. At the genus level, *Cricotopus* and *Rheocricotopus* were the representative taxa.

#### 3.2.2. Spatiotemporal Distribution of Taxonomic Composition

**Figure 2:**
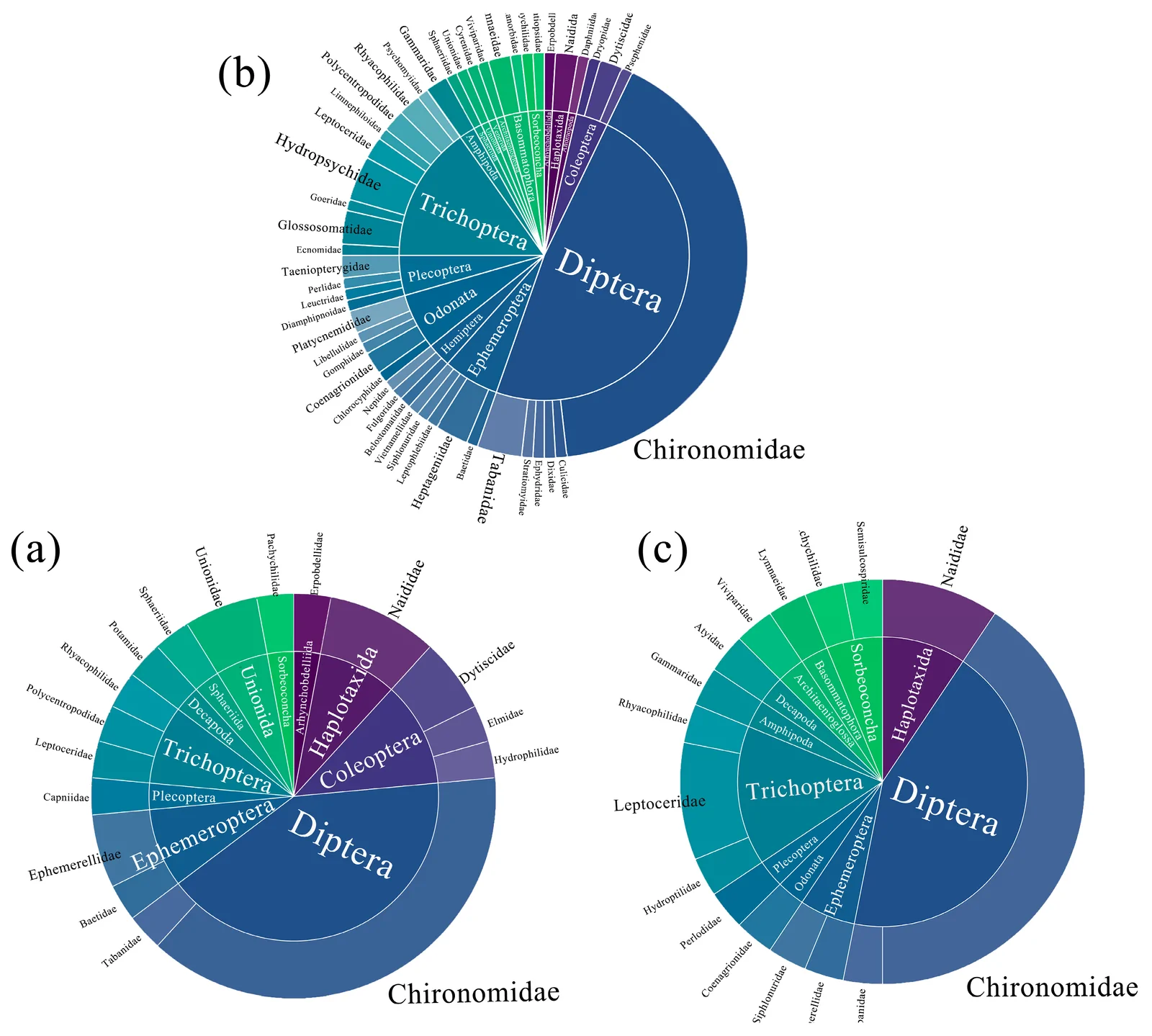
Taxonomic composition of the benthic invertebrate community in the Hun River Basin across seasons. (**a**) Spring, (**b**) Summer, (**c**) Autumn. The inner ring colors indicate different taxonomic orders, while the text labels on the outer ring correspond to the respective families.

Community composition varied among seasons at the order and family taxonomic levels (Figure 2). In spring, a total of 3 phyla, 6 classes, 11 orders, 17 families, and 33 genera were recorded; in summer, 3 phyla, 7 classes, 17 orders, 47 families, and 85 genera were recorded; and in autumn, 3 phyla, 4 classes, 11 orders, 16 families, and 29 genera were recorded. Overall, the benthic invertebrate community in spring was predominantly composed of Chironomidae (Diptera). In summer, the taxon richness of the benthic invertebrate community increased markedly, with Hemiptera and Odonata newly appearing within Insecta, and four additional dipteran families (Culicidae, Dixidae, Ephydridae, and Stratiomyidae) being recorded. Concurrently, several new families were detected within Ephemeroptera, Plecoptera, and Trichoptera. The benthic invertebrate community exhibited its highest diversity during summer. The community composition in autumn was generally similar to that in spring. However, the taxon richness of Leptoceridae and Naididae increased relative to spring, with Chironomidae as the consistently dominant taxon. Notably, the EPT taxa (Ephemeroptera, Plecoptera, and Trichoptera) along with Diptera dominated the community, accounting for 64.71%, 74.18%, and 68.75% of the total taxa across the three seasons, respectively. Among these, Chironomidae was the dominant taxon across the three seasons, representing 38.24%, 41.07%, and 40.63% of the total taxa in spring, summer, and autumn, respectively.

**Figure 3:**
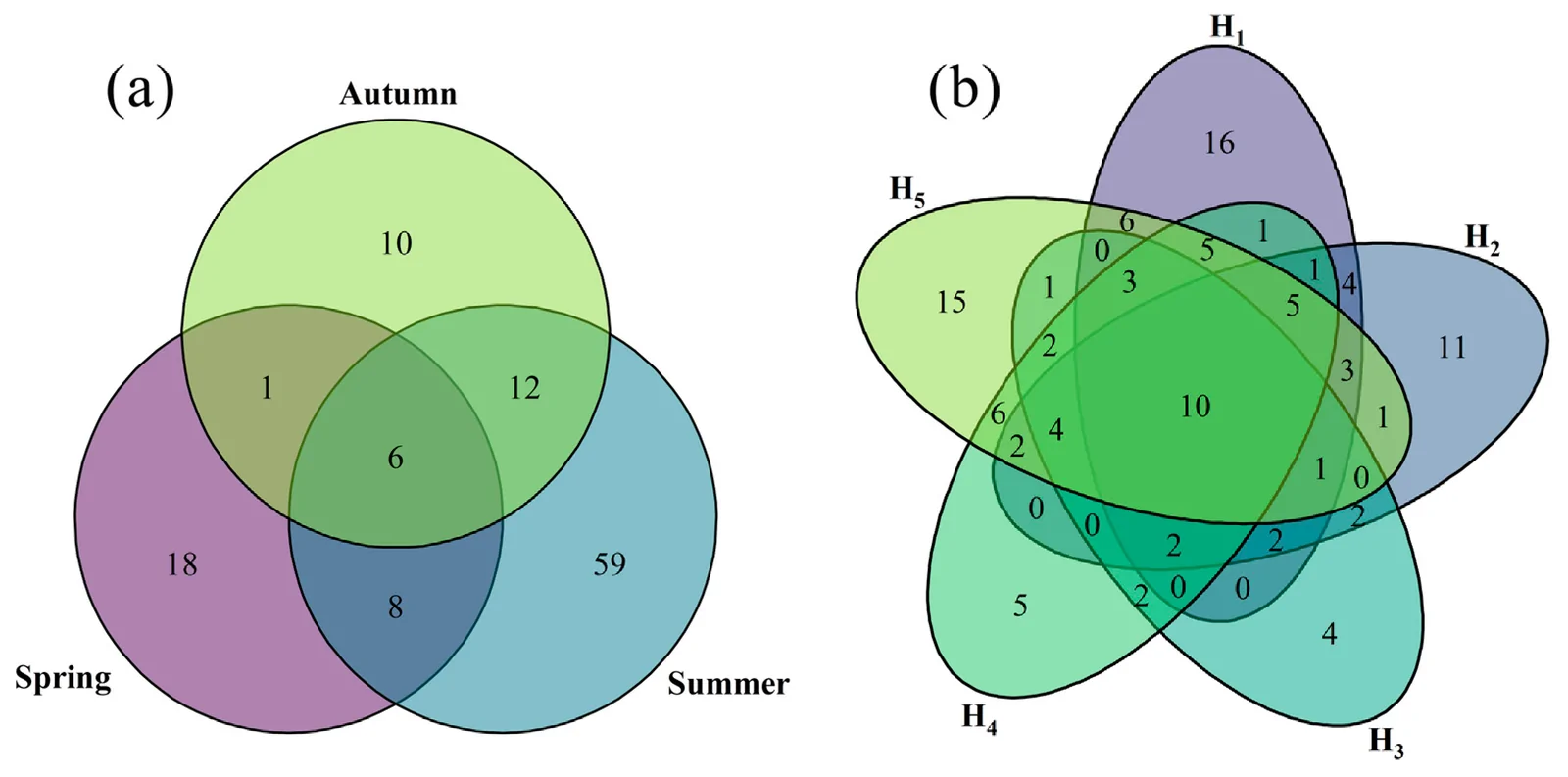
Venn diagrams showing the genus-level community composition of benthic invertebrates in the Hun River basin. (**a**) Among different seasons. (**b**) Among different sampling sites.

To elucidate the spatiotemporal distribution characteristics of the community composition at the genus level, Venn diagrams were constructed based on both temporal and spatial dimensions. At the temporal (seasonal) scale (Figure 3a), a total of 114 genera were detected across spring, summer, and autumn. Among these, 6 genera were shared across all three seasons: *Nais*, *Chironomus*, *Paratanytarsus*, *Chrysops*, *Rhyacophila*, and *Sulcospira*. The numbers of season-specific genera were 18 for spring, 59 for summer, and 10 for autumn. The numbers of shared genera between spring–summer, spring–autumn, and summer–autumn were 14, 7, and 18, respectively. At the spatial scale (Figure 3b), 10 genera were found to be common to all sampling sites: *Chironomus*, *Limnophyes*, *Orthocladius*, *Propsilocerus*, *Stictochironomus*, *Tenuibaetis*, *Glossosoma*, *Hydromanicus*, *Polyplectropus*, and *Sulcospira*. Site H5 contained the highest number of genera (64), while site H3 exhibited the lowest (33). The counts of site-specific genera for H1, H2, H3, H4, and H5 were 16, 11, 4, 5, and 15, respectively.

### 3.3. Spatiotemporal Patterns of Community Relative Abundance and Dominant Genera

**Figure 4:**
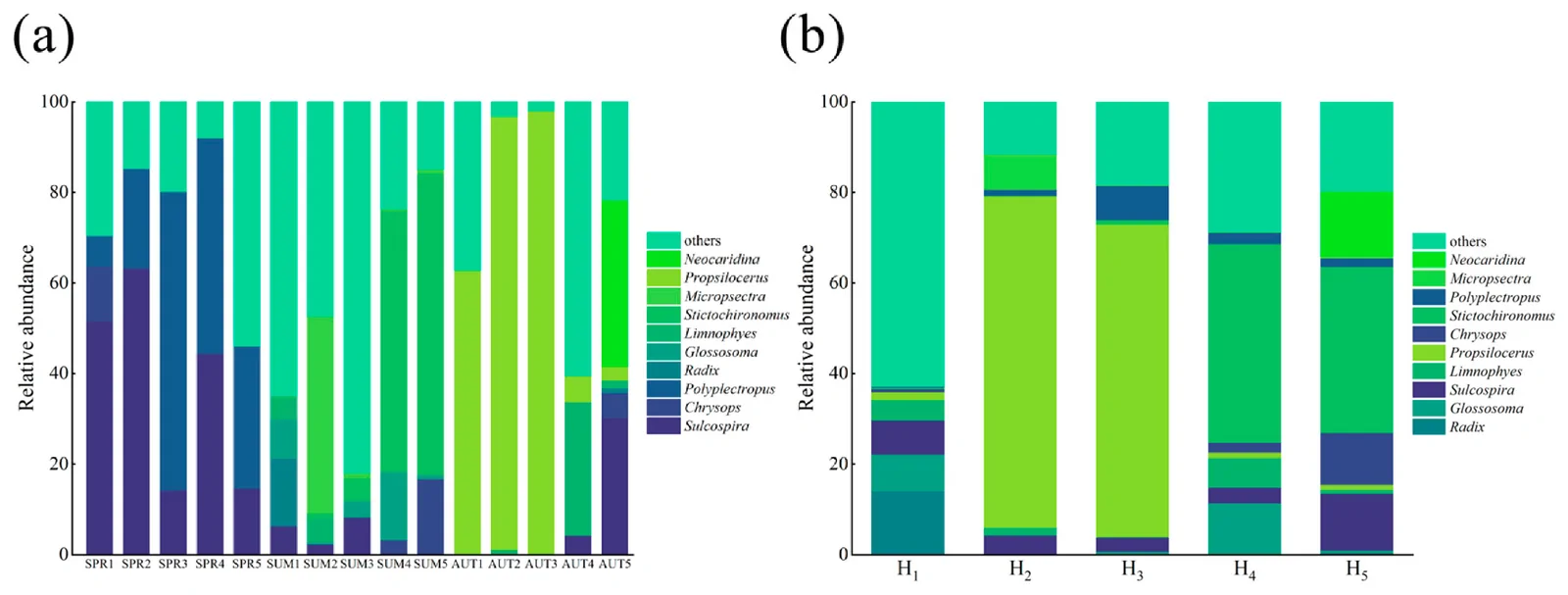
Spatiotemporal patterns of dominant genera in benthic invertebrate communities based on relative sequence abundance. (**a**) All season-site combinations. (**b**) Spatial scale. H1–H5 represent the same site sampled across different seasons, i.e., H1 comprises samples SPR1, SUM1, and AUT1; H2 comprises samples SPR2, SUM2, and AUT2; H3 comprises samples SPR3, SUM3, and AUT3; H4 comprises samples SPR4, SUM4, and AUT4; and H5 comprises samples SPR5, SUM5, and AUT5.

Genus-level relative sequence abundance analysis was conducted based on the read counts of OTUs assigned to the same genus. It should be noted that the genus-level taxonomic assignments and relative sequence abundances reported herein represent operational classifications derived from sequence clustering, rather than absolute species counts or definitive taxonomic identities. Furthermore, the characterization of dominant genera was performed within the context of the reference database coverage. In detail, the top 10 dominant genera of benthic invertebrates in the study basin were *Propsilocerus* (36.17%), *Stictochironomus* (14.00%), *Sulcospira* (6.74%), *Neocaridina* (3.83%), *Chrysops* (3.37%), *Micropsectra* (3.15%), *Glossosoma* (2.46%), *Radix* (2.21%), *Limnophyes* (2.17%), and *Polyplectropus* (2.05%). In total, these 10 dominant genera accounted for 76.16% of the total relative sequence abundance, constituting the principal structural taxa of the community (Figure 4a). Among them, 7 dominant genera belonged to the class Insecta, collectively contributing 63.37% to the total relative sequence abundance, and 4 genera belonged to the family Chironomidae, contributing 55.50%. *Sulcospira* was the most widespread dominant genus and was recorded at 11 season-site combinations across the three seasons and five sites.

At the temporal (seasonal) scale, the dominant genera in the studied watershed communities varied considerably. In spring (SPR1–SPR5), *Sulcospira* and *Polyplectropus* were the predominant genera, each accounting for over 46% at all five sites. Specifically, *Sulcospira* comprised >50% at SPR1 (51.60%) and SPR2 (63.27%), whereas *Polyplectropus* peaked at SPR3 (65.85%). Together, these two genera represented over 80% of the total relative sequence abundance at SPR2–SPR4, with *Chrysops* contributing an additional 12% at SPR1. In summer (SUM1–SUM5), the generic composition became markedly more heterogeneous. The relative sequence abundance of *Sulcospira* declined sharply at SUM1 (6.16%), SUM2 (2.16%), SUM3 (8.25%), and SUM4 (0.37%). In contrast, *Glossosoma* exhibited a peak at SUM4 (15.27%) and SUM1 (8.6%), while remaining below 5% at the other three sites. *Limnophyes* maintained low relative sequence abundances across all summer sites (0.25–5.16%). Notably, community structure at SUM4 and SUM5 was dictated by *Stictochironomus*, which constituted 59% and 66.41% of the communities, respectively—far exceeding its proportions at SUM1–SUM3 (0.35–4.57%). *Micropsectra* dominated SUM2 (43.41%) but was nearly absent elsewhere (<2%). *Chrysops* peaked at SUM5 (16.72%), and *Radix* reached 14.91% at SUM1. Due to this high compositional complexity, the “others” fraction remained substantial in SUM1, SUM2, and SUM3, each exceeding 47%. In autumn (AUT1–AUT5), *Propsilocerus* was the core dominant genus, occupying 62.8%, 95.69%, and 97.99% at AUT1–AUT3, but dropping to 5.82% and 3.01% at AUT4 and AUT5. Meanwhile, *Sulcospira* was detected only at AUT4 (4.25%) and AUT5 (30.18%), *Limnophyes* reached 29.42% at AUT4, and *Neocaridina* accounted for 36.84% at AUT5.

Integrating data collected over three seasons from the same sites further revealed (Figure 4b) that the composition of dominant genera varied among sites. At H1, the relative sequence abundances of dominant genera exhibited a fairly even distribution, with the dominant genera being *Radix* (13.98%), *Glossosoma* (8.07%), and *Sulcospira* (7.54%). The community structure at H2 was similar to that at H3, with *Propsilocerus* as the core dominant genus, representing 73.11% and 69.13% of the relative sequence abundance, respectively. Conversely, the community structures at H4 and H5 were characterized by *Stictochironomus* as the core dominant genus, representing 43.81% and 36.66%, respectively. Furthermore, H5 exhibited the highest richness of dominant genera at the spatial scale, and *Propsilocerus* and *Stictochironomus* (Chironomidae) were the core dominant genera at site clusters H2/H3 and H4/H5, respectively.

### 3.4. Benthic Invertebrate Community Diversity Based on eDNA Metabarcoding

#### 3.4.1. Alpha Diversity Across Temporal and Spatial Scales

**Figure 5:**
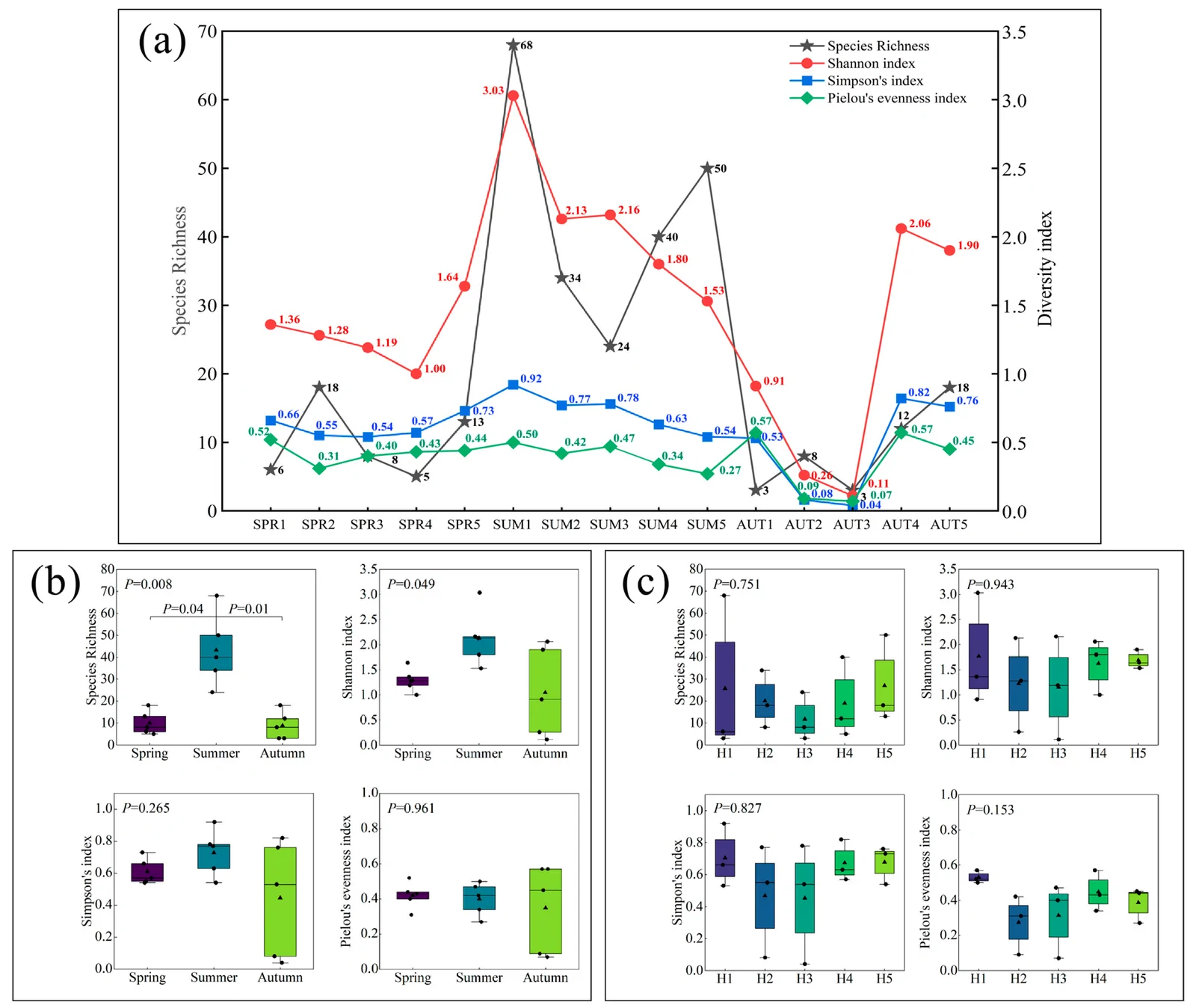
Alpha diversity indices and differential significance tests for benthic invertebrates in the Hun River basin. (**a**) Alpha diversity indices at each season-site combination. (**b**) Tests for differences in alpha diversity at the temporal (seasonal) scale. (**c**) Tests for differences in alpha diversity at the spatial scale.

In this study, alpha diversity indices (Richness, Shannon index, Simpson index, and Pielou’s evenness index) were calculated to evaluate the community diversity (Figure 5a). These indices were based on relative sequence abundances of OTUs and served as proxies for community-level compositional and structural attributes, rather than as precise measures of absolute abundance or biomass [37]. The results showed that Richness exhibited significant overall seasonal variation (*p* = 0.008), while the Shannon index was significant (*p* = 0.049). Multiple comparisons adjustment indicated significant differences in Richness between spring and summer (*p* = 0.04) and between summer and autumn (*p* = 0.01). However, the Shannon index did not differ significantly in any seasonal comparisons. Additionally, neither the Simpson index nor the Pielou’s evenness index displayed significant seasonal changes (Figure 5b). Subsequent significance tests for spatial differences across the five sampling sites revealed no significant variations in Richness (*p* = 0.751), Shannon index (*p* = 0.943), Simpson index (*p* = 0.827), or Pielou’s evenness index (*p* = 0.153) (Figure 5c).

#### 3.4.2. Community Beta Diversity and Spatiotemporal Differentiation

**Figure 6:**
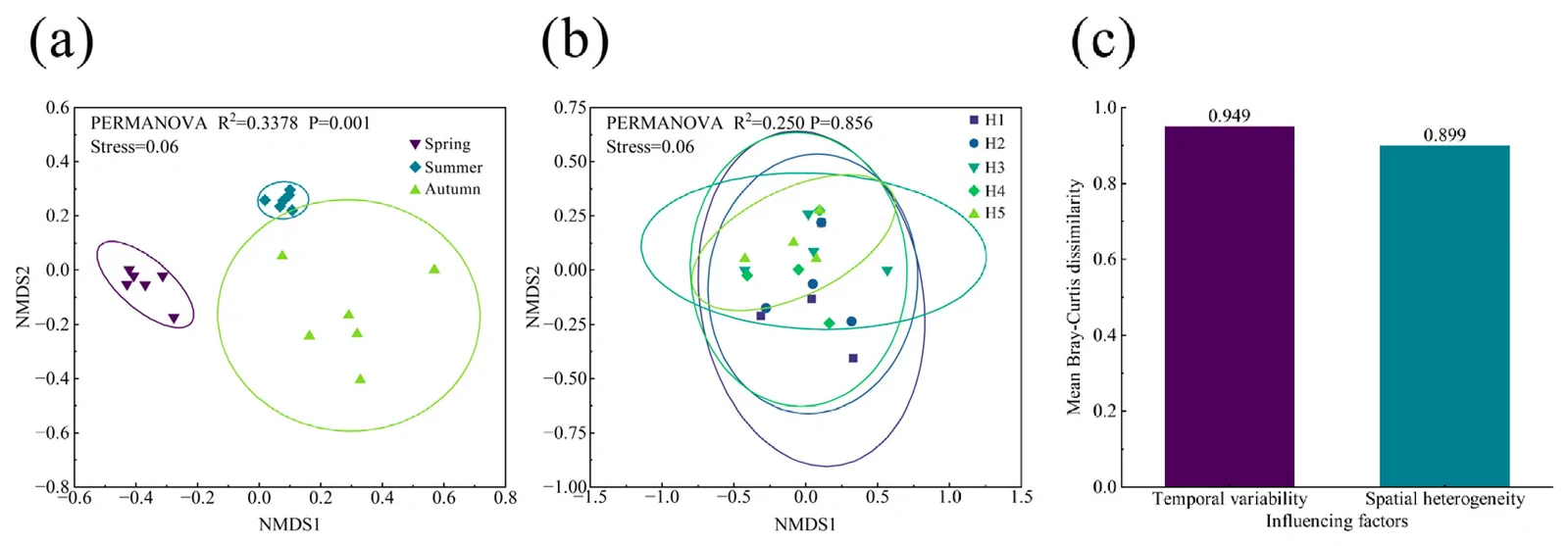
Beta diversity and community structural dissimilarity across spatiotemporal scales. (**a**) Beta diversity at the temporal (seasonal) scale. (**b**) Beta diversity at the spatial scale. (**c**) Community structural dissimilarity across spatiotemporal scales.

Based on the Bray–Curtis dissimilarity matrix, non-metric multidimensional scaling (NMDS) was used to visualize community beta diversity, combined with PERMDISP and PERMANOVA to quantitatively evaluate the explanatory power of temporal and spatial scales on community structural variation. The NMDS ordination yielded a stress value of 0.06 (<0.1), which indicates a satisfactory fit and high reliability of the ordination analysis (Figure 6a). At the temporal (seasonal) scale, samples from spring (SPR1–SPR5), summer (SUM1–SUM5), and autumn (AUT1–AUT5) displayed clear separation. The global PERMDISP test was significant (F = 8.763, *p* = 0.010), and PERMANOVA revealed a highly significant statistical effect of season on community structure (R^2^ = 0.3378, *p* = 0.001, permutations = 999), confirming substantial seasonal differentiation. Subsequent pairwise post hoc multiple comparisons indicated significant compositional differentiations between spring–summer (F = 3.962, R^2^ = 0.331, *p* = 0.024), spring–autumn (F = 3.724, R^2^ = 0.318, *p* = 0.021), and summer–autumn (F = 1.904, R^2^ = 0.192, *p* = 0.030). Pairwise PERMDISP tests revealed significant differences in community dispersion between spring and summer (F = 9.110, *p* = 0.015) and between spring and autumn (F = 18.513, *p* = 0.007), but not between summer and autumn (F = 0.306, *p* = 0.577). These findings suggest that the intergroup differentiations between spring–summer and spring–autumn were jointly driven by centroid shifts and differences in within-group dispersion, whereas the significant differentiation between summer and autumn was solely attributable to shifts in community centroids. At the spatial scale, samples exhibited substantial overlap in their distribution (Figure 6b). Although spatial grouping explained 25% of the total variation, both the global PERMDISP (F = 0.483, *p* = 0.778) and PERMANOVA (F = 0.834, R^2^ = 0.250, *p* = 0.856) were far from reaching significance.

In addition, we calculated the mean Bray–Curtis dissimilarity for the corresponding seasonal and spatial beta diversity (Figure 6c). The results showed that the temporal beta diversity of the community in the study watershed was 0.949, and the spatial beta diversity was 0.899, both at relatively high levels.

### 3.5. Contributing Genera to Spatiotemporal Variation in the Benthic Invertebrate Community

To further elucidate the contribution of the key genera to community dissimilarity, similarity percentage analysis (SIMPER) was employed to describe the contributions of the top 10 genera accounting for spatiotemporal variations (Table 1). At the temporal (seasonal) scale, *Stictochironomus*, *Micropsectra*, and *Polyplectropus* exhibited the highest contribution rates (ranked top three) to community dissimilarity between spring and summer, accounting for 24.3%, 8.2%, and 5.9%, respectively, with the cumulative contribution of the top 10 genera reaching 67.5%. For the summer–autumn comparison, *Propsilocerus*, *Stictochironomus*, and *Micropsectra* were the dominant contributors, with rates of 27.3%, 16.8%, and 5.3%, respectively, and the cumulative contribution of the top 10 genera was 72.8%. In the spring–autumn comparison, *Propsilocerus*, *Sulcospira*, and *Polyplectropus* were the primary contributors, with contribution rates of 41.9%, 13.6%, and 9.5%, respectively, and the cumulative contribution of the top 10 genera reached 87.8%, which was the highest among the three seasonal comparisons.

At the spatial scale, for the H1–H2 and H1–H3 comparisons, community dissimilarity was primarily attributed to *Propsilocerus*, with contribution rates of 31.8% and 28.9%, respectively. In contrast, *Stictochironomus* was the dominant contributor for the H1–H4 and H1–H5 comparisons, accounting for 16.4% and 19.7%, respectively. Among all pairwise site comparisons, *Propsilocerus* exhibited the highest contribution to the H2–H3 dissimilarity (45.8%). For the H2–H3 comparison, the cumulative contribution of the top 6 genera reached 81.5%, and that of the top 10 genera reached 87.7%. *Propsilocerus* was consistently the dominant contributor between H2 and each of the other sites. In the H3–H4 comparison, the top three contributors were *Propsilocerus*, *Stictochironomus*, and *Polyplectropus*, accounting for 28.1%, 16.1%, and 10.2%, respectively. For the H3–H5 comparison, the primary contributors were *Stictochironomus* (20.5%), *Propsilocerus* (19.0%), and *Neocaridina* (10.5%). Finally, in the H4–H5 comparison, the dominant contributors were *Stictochironomus* (28.5%), *Neocaridina* (11.4%), and *Sulcospira* (10.4%). Overall, *Propsilocerus* and *Stictochironomus* exhibited relatively high contribution rates at the spatial scale.

## 4. Discussion

### 4.1. Performance of eDNA Metabarcoding in Monitoring Benthic Invertebrate Communities in the Hun River Basin

#### 4.1.1. Comparison of eDNA Metabarcoding and Conventional Survey Methods

In this study, eDNA metabarcoding was employed to monitor benthic invertebrate diversity in the Hun River Basin (middle and upper reaches). The number of detected taxa surpassed that obtained from previous conventional surveys [24,25,26,27,28,29,30]. Specifically, in 2012, Meng et al. established 65 sampling sites across the Huntai River Basin (Hun River and Taizi River) and conducted quantitative collections using Surber nets, yielding a total of 71 genera of benthic invertebrates, belonging to 4 phyla, 7 classes, 13 orders, and 35 families [29]. Based on morphological identification from surveys of the Hun River conducted in April and May 2012, Han et al. recorded 48 species of benthic invertebrates, belonging to 4 phyla, 7 classes, 13 orders, and 35 families [26]. In 2016, Li conducted quantitative sampling in the Dahuntai Basin (Daliao River, Hun River, and Taizi River) and identified 90 species of benthic invertebrates, representing 3 phyla, 7 classes, 12 orders, 25 families, and 62 genera [30]. In the present study, eDNA metabarcoding detected a total of 3 phyla, 7 classes, 18 orders, 56 families, 114 genera, and 161 species of benthic invertebrates in the Hun River Basin. Among these, 64 operational taxonomic units (OTUs) were assigned to the family Chironomidae, representing a significant improvement in taxonomic resolution at the generic level compared with traditional morphological methods.

#### 4.1.2. Core Advantages and Application Potential of eDNA Metabarcoding

In this study, eDNA metabarcoding exhibited the following strengths. First, it successfully detected taxa that are often missed by conventional netting, such as Naididae and Daphniidae. Second, it circumvented sampling biases caused by the escape of highly mobile individuals due to water disturbance, enabling the detection of fast-moving groups including Dytiscidae and Gammaridae. Third, it reduced morphological identification errors arising from differences in developmental stages and life-history rhythms. For aquatic insect groups that are morphologically complex and highly similar in appearance, eDNA achieved precise identification to the genus level [38]. Finally, eDNA metabarcoding substantially reduced the time and labor costs associated with sampling, minimized the workload of field collection and laboratory sorting, and lessened dependence on expert taxonomic expertise, thereby ensuring timely data processing for subsequent large-scale analyses. Our results provide evidence supporting the potential applicability of eDNA metabarcoding for monitoring benthic invertebrates in the Hun River Basin.

#### 4.1.3. Limitations and Future Perspectives for eDNA Metabarcoding

However, this study identified certain limitations associated with this approach. Although the sampling period has encompassed spring, summer, and autumn, and the layout of sampling sites has comprehensively considered anthropogenic activities and watershed environment, the detection efficiency of eDNA samples is still constrained by factors such as sampling timing, number of sites, and frequency. Therefore, a single round of periodic monitoring and survey cannot fully represent the community diversity, highlighting the critical need to establish systematic and long-term eDNA biomonitoring frameworks to comprehensively elucidate the benthic invertebrate communities in the target watershed.

Subsequently, in the PCR amplification process of this study, the universal primers mlCOIintF and dgHCO2198 were selected for their specific design targeting the conserved regions of the mitochondrial COI gene in Metazoa. These optimized short-fragment primers enable the simultaneous capture of the majority of taxa within benthic invertebrate communities in the reaction [33]. This primer set enhances the PCR success rate for partially degraded eDNA samples and those containing inhibitors such as humic acids [39]. However, this primer set exhibits a clear bias toward arthropod taxa, with limited amplification efficiency for members of Annelida, Platyhelminthes, and certain crustacean groups. This bias partly explains the discrepancy between our results and those of Zhang et al. [24], who conducted a morphology-based survey of benthic macroinvertebrates in the middle reaches of the Hun River and reported a different oligochaete species composition. It is worth noting that the successful detection of Naididae in this study provides a reliable basis for inferring the presence or absence of this taxonomic group. Nevertheless, the potential distorting effects of primer bias on relative sequence abundance estimates should not be overlooked. As such, the obtained sequence abundances of Naididae are indicative of sequencing signal intensity only and should not be equated with genuine taxonomic ratios.

Additionally, the application of eDNA metabarcoding for monitoring aquatic biodiversity is constrained by the limitations of reference databases. The quality and completeness of these databases determine the reliability and accuracy of species identification when employing eDNA metabarcoding [12,14,40]. In this study, species annotation was performed against the Chinese Freshwater Macroinvertebrate Barcode Database (which can be cross-referenced with international public databases such as GenBank and BOLD) [40]. However, as this database is still under continuous improvement, data for certain taxonomic groups (e.g., Oligochaeta) remain incomplete and require further supplementation, thereby limiting the comprehensiveness of annotation [40]. This inherent data gap constitutes the principal factor underlying the incomplete taxonomic annotation observed herein, and directly explains why a subset of OTUs could not be confidently assigned or could only be resolved at coarser taxonomic ranks. Thus, to support benthic biodiversity conservation and freshwater ecological health assessment via eDNA metabarcoding, it is imperative to continuously expand and refine the reference database, thereby reinforcing the data foundation and ensuring robust technical safeguards.

### 4.2. Benthic Invertebrate Diversity and Its Spatiotemporal Variations in the Hun River Basin

#### 4.2.1. Diversity of Dominant Benthic Invertebrate Taxa

The results of this study are comparable to the benthic invertebrate community compositions observed in typical freshwater ecosystems within China, such as the Huangshui River [41], Jinsha River [42,43], and Beijing Yongding River basins [44]. Based on the relative sequence abundance, the top 10 dominant genera were all common benthic invertebrate taxa in local freshwater ecosystems. Among these, seven genera belonged to the class Insecta, accounting for 63.37% of the total relative sequence abundance. Within these seven, four belonged to the family Chironomidae, contributing 55.50% of the total. Additionally, *Sulcospira* was the most widely distributed dominant genus. In this study, the species richness and relative sequence abundance of Chironomidae were higher than those of all other taxa. This phenomenon is closely linked to its life cycle, reproductive strategies, and broad physiological adaptability [45,46]. Moreover, certain chironomid larvae can thrive in polluted aquatic environments, exhibiting considerable tolerance to contamination [47]. For instance, the abundances of *Chironomus kiiensis*, *Chironomus javanus*, and *Polypedilum trigonus* are positively correlated with elevated concentrations of organic matter and heavy metal ions in the sediment [48]. Furthermore, the genus *Neocaridina* represents a predominant taxon within the study watershed. Its feeding behavior facilitates the decomposition of organic matter on the riverbed and accelerates nutrient cycling (e.g., nitrogen and phosphorus), thereby playing a crucial role in maintaining aquatic ecosystem equilibrium. This study reveals that the genus *Sulcospira*, a viviparid freshwater snail, exhibits a widespread distribution pattern in the Hun River basin. This pattern likely arises not from stochastic dispersal events but may instead be associated with intrinsic biological traits and environmental selection pressures. Nevertheless, given that freshwater snails serve as intermediate hosts for trematodes, which can diminish agricultural yields and transmit diseases to humans and livestock, their surveillance and management should be prioritized [49].

#### 4.2.2. Ecological Interpretation of Seasonal and Spatial Patterns

This study indicates that the diversity of benthic invertebrates in the Hun River basin exhibits temporal (seasonal) variation. Richness showed significant seasonal variation (*p* = 0.008), whereas the Shannon index was significant (*p* = 0.049). Based on previous studies, it is speculated that this change may be related to alterations in hydrological factors associated with seasonal succession [50]. Generally, summer is characterized by suitable water temperatures and sufficient sunlight, which promote phytoplankton proliferation. This process augments food availability for benthic invertebrates while simultaneously promoting the growth and reproduction of aquatic insects. In contrast, spring and autumn exhibit lower water temperatures and consequently reduced energy flow, thereby favoring only cold-tolerant and hypoxia-tolerant taxa (e.g., *Propsilocerus*, *Sulcospira*). Furthermore, beta diversity assessed at the seasonal scale revealed that seasonality had a highly significant influence on community structure. Spatial scale analysis revealed certain differences in community composition and structure. However, neither alpha nor beta diversity exhibited statistically significant variation.

In this study, Plecopteran insects (e.g., Perlidae, Capniidae), which exhibit low environmental tolerance and depend on clean water [51], were exclusively detected at sites H1, H4, and H5. These sites are located in the upper reaches or in river sections subject to relatively weak human interference, where water clarity is comparatively higher, which may have provided favorable conditions for these sensitive taxa. Conversely, Trichoptera, which are sensitive to dissolved oxygen and pH, were found in substantial numbers at all sites except H3, which experiences pronounced anthropogenic stress [52,53]. SIMPER analysis indicated that chironomid larvae with different pollution tolerances exhibited relatively high contributions to the observed dissimilarity at the spatial scale. Specifically, the dominant contributor genus between H1 and H2/H3 was *Propsilocerus*, whereas that between H1 and H4/H5 was *Stictochironomus*. These patterns may be associated with changes in water quality along the urban gradient [54] or with the regulation of hydraulic engineering projects within the watershed [55]. However, the aforementioned inferences regarding the underlying mechanisms of spatiotemporal variation are not supported by measured environmental covariates, and are considered only as plausible hypotheses that await further testing. In subsequent studies, we will investigate the correlations between benthic invertebrate communities and seasonal and site-specific water environmental parameters.

## 5. Conclusions

In this study, environmental DNA (eDNA) metabarcoding was employed to reveal the diversity and spatiotemporal variations in benthic invertebrates in the Hun River basin (the middle and upper reaches). A total of 3 phyla, 7 classes, 18 orders, 56 families, 114 genera, and 161 species were detected. The community composition exhibited variations across both seasonal and spatial scales, as reflected by differences in OTU richness, dominant taxa and their relative sequence abundances, and alpha diversity indices. PERMDISP and PERMANOVA revealed that season had a highly significant effect on community structure, whereas the differences among spatial groups were not significant. SIMPER analysis identified the key genera that contributed substantially to community dissimilarity among sampling groups, including *Propsilocerus*, *Stictochironomus*, *Tanytarsus*, *Neocaridina*, etc. This study confirmed the potential applicability of eDNA metabarcoding for spatiotemporal monitoring of benthic invertebrates in typical freshwater ecosystems such as urban rivers, while also exposing several current limitations in the application of this technique. Overall, this study provides fundamental data for aquatic biodiversity conservation and water ecological health assessment in the Hun River basin, while also serving as a regional reference case for improving eDNA-based aquatic biomonitoring protocols.

## Figures and Tables

**Table 1:** Major contributing genera to observed spatiotemporal dissimilarity in benthic invertebrate communities.

Category	Genus-Level Taxon	Individual Contribution (%)	Cumulative Contribution (%)
SPR–SUM	*Stictochironomus*	24.3%	24.3%
	*Micropsectra*	8.2%	32.5%
	*Polyplectropus*	5.9%	38.4%
	*Unio*	5.8%	44.2%
	*Glossosoma*	5.1%	49.3%
	*Sulcospira*	5.1%	54.4%
	*Chrysops*	4.0%	58.4%
	*Hydromanicus*	3.5%	61.9%
	*Radix*	2.8%	64.7%
	*Acricotopus*	2.8%	67.5%
SUM–AUT	*Propsilocerus*	27.3%	27.3%
	*Stictochironomus*	16.8%	44.1%
	*Micropsectra*	5.3%	49.4%
	*Sulcospira*	4.8%	54.2%
	*Neocaridina*	4.4%	58.6%
	*Glossosoma*	3.4%	62.0%
	*Chrysops*	3.2%	65.2%
	*Unio*	3.1%	68.3%
	*Hydromanicus*	2.3%	70.6%
	*Radix*	2.2%	72.8%
SPR–AUT	*Propsilocerus*	41.9%	41.9%
	*Sulcospira*	13.6%	55.5%
	*Polyplectropus*	9.5%	65.0%
	*Neocaridina*	6.8%	71.8%
	*Limnophyes*	4.4%	76.2%
	*Nais*	3.7%	79.9%
	*Tanytarsus*	2.6%	82.5%
	*Paratanytarsus*	2.4%	84.9%
	*Polypedilum*	1.5%	86.4%
	*Chrysops*	1.4%	87.8%
H1–H2	*Propsilocerus*	31.8%	31.8%
	*Sulcospira*	13.0%	44.8%
	*Micropsectra*	11.4%	56.2%
	*Polyplectropus*	4.6%	60.8%
	*Hydromanicus*	4.5%	65.3%
	*Radix*	3.3%	68.6%
	*Tinodes*	3.0%	71.6%
	*Hippeutis*	2.4%	74.0%
	*Acricotopus*	2.4%	76.4%
	*Limnophyes*	2.2%	78.6%
H1–H3	*Propsilocerus*	28.9%	28.9%
	*Polyplectropus*	11.7%	40.6%
	*Unio*	9.0%	49.6%
	*Sulcospira*	5.3%	54.9%
	*Radix*	4.4%	59.3%
	*Acricotopus*	3.9%	63.2%
	*Tinodes*	3.8%	67.0%
	*Hippeutis*	3.3%	70.3%
	*Spirosperma*	3.2%	73.5%
	*Glossosoma*	3.1%	76.6%
H1–H4	*Stictochironomus*	16.4%	16.4%
	*Sulcospira*	7.2%	23.6%
	*Limnophyes*	7.1%	30.7%
	*Polyplectropus*	5.9%	36.6%
	*Glossosoma*	5.7%	42.3%
	*Propsilocerus*	5.7%	48.0%
	*Nais*	5.6%	53.6%
	*Radix*	4.8%	58.4%
	*Tinodes*	4.2%	62.6%
	*Hippeutis*	3.5%	66.1%
H1–H5	*Stictochironomus*	19.7%	19.7%
	*Neocaridina*	10.4%	30.1%
	*Sulcospira*	10.4%	40.5%
	*Tanytarsus*	7.6%	48.1%
	*Chrysops*	6.7%	54.8%
	*Polyplectropus*	6.2%	61.0%
	*Radix*	3.7%	64.7%
	*Tinodes*	3.1%	67.8%
	*Hippeutis*	2.6%	70.4%
	*Acricotopus*	2.4%	72.8%
H2–H3	*Propsilocerus*	45.8%	45.8%
	*Micropsectra*	11.6%	57.4%
	*Sulcospira*	10.8%	68.2%
	*Polyplectropus*	4.7%	72.9%
	*Hydromanicus*	4.7%	77.6%
	*Unio*	3.9%	81.5%
	*Limnophyes*	1.8%	83.3%
	*Euryhapsis*	1.6%	84.9%
	*Vietnamella*	1.4%	86.3%
	*Spirosperma*	1.4%	87.7%
H2–H4	*Propsilocerus*	32.0%	32.0%
	*Sulcospira*	11.7%	43.7%
	*Micropsectra*	11.7%	55.4%
	*Stictochironomus*	10.2%	65.6%
	*Hydromanicus*	4.8%	70.4%
	*Polyplectropus*	3.8%	74.2%
	*Limnophyes*	3.0%	77.2%
	*Glossosoma*	2.7%	79.9%
	*Nais*	2.4%	82.3%
	*Euryhapsis*	1.7%	84.0%
H2–H5	*Propsilocerus*	28.1%	28.1%
	*Stictochironomus*	15.5%	43.6%
	*Sulcospira*	9.9%	53.5%
	*Micropsectra*	8.1%	61.6%
	*Neocaridina*	7.6%	69.2%
	*Chrysops*	5.0%	74.2%
	*Hydromanicus*	3.3%	77.5%
	*Tanytarsus*	3.1%	80.6%
	*Polyplectropus*	1.8%	82.4%
	*Polypedilum*	1.6%	84.0%
H3–H4	*Propsilocerus*	28.1%	28.1%
	*Stictochironomus*	16.1%	44.2%
	*Polyplectropus*	10.2%	54.4%
	*Unio*	8.2%	62.6%
	*Limnophyes*	5.0%	67.6%
	*Glossosoma*	4.5%	72.1%
	*Nais*	4.2%	76.3%
	*Spirosperma*	2.9%	79.2%
	*Sulcospira*	2.1%	81.3%
	*Paratanytarsus*	1.6%	82.9%
H3–H5	*Stictochironomus*	20.5%	20.5%
	*Propsilocerus*	19.0%	39.5%
	*Neocaridina*	10.5%	50.0%
	*Sulcospira*	9.5%	59.5%
	*Chrysops*	6.6%	66.1%
	*Tanytarsus*	5.8%	71.9%
	*Polyplectropus*	5.1%	77.0%
	*Unio*	4.2%	81.2%
	*Polypedilum*	2.2%	83.4%
	*Spirosperma*	1.7%	85.1%
H4–H5	*Stictochironomus*	28.5%	28.5%
	*Neocaridina*	11.4%	39.9%
	*Sulcospira*	10.4%	50.3%
	*Chrysops*	7.2%	57.5%
	*Tanytarsus*	7.2%	64.7%
	*Polyplectropus*	4.9%	69.6%
	*Limnophyes*	3.2%	72.8%
	*Glossosoma*	3.1%	75.9%
	*Nais*	2.7%	78.6%
	*Polypedilum*	2.6%	81.2%

Note: SPR consists of samples SPR1 to SPR5; SUM consists of samples SUM1 to SUM5; AUT consists of samples AUT1 to AUT5; H1 consists of samples SPR1, SUM1, and AUT1; H2 consists of samples SPR2, SUM2, and AUT2; H3 consists of samples SPR3, SUM3, and AUT3; H4 consists of samples SPR4, SUM4, and AUT4; and H5 consists of samples SPR5, SUM5, and AUT5.

## Data Availability

The raw data supporting the conclusions of this article is deposited in BioProject: PRJNA1510895. For further inquiries, please contact the authors.

## Supplementary Materials

The following supporting information can be downloaded at: https://www.mdpi.com/article/10.3390/biology15181597/s1, Table S1: Basic information on alignment-based annotation of OTUs for benthic invertebrates in the Hun River basin; Table S2: Checklist of taxonomically annotated taxa for benthic invertebrates in the Hun River basin.
