# Supplementary material for: Environmental DNA Metabarcoding Reveals Diversity and Spatiotemporal Variations of Benthic Invertebrates in Hun River Basin, China
Source: Biology (Basel). 2026 Sep 10;15(18):1597. doi: 10.3390/biology15181597 (PMC13604782; doi:10.3390/biology15181597)
Supplement: Supplementary file 1 [file biology-15-01597-s001.zip › Table S1. Basic information on alignment-based annotation of OTUs for benthic invertebrates in the Hun River basin.pdf]

Table S1. Basic information on alignment-based annotation of OTUs for benthic invertebrates in the Hun River basin.

**Samples**		**OTU**	**Phylum**	**Class**	**Order**	**Family**	**Genus**	**Species**
H1	SPR	6	2	2	4	6	6	6
	SUM	68	3	7	14	36	55	68
	AUT	3	1	1	2	2	3	3
	Total	75	3	7	14	38	59	75
H2	SPR	18	3	6	9	11	17	18
	SUM	34	3	3	8	16	30	34
	AUT	8	2	2	2	2	8	8
	Total	55	3	6	12	22	48	55
H3	SPR	8	2	2	5	6	8	8
	SUM	24	3	6	11	15	24	24
	AUT	3	2	2	2	2	3	3
	Total	34	3	6	12	18	33	34
H4	SPR	5	2	2	4	4	5	5
	SUM	40	3	6	12	22	37	40
	AUT	12	3	3	5	7	12	12
	Total	53	3	6	13	26	48	53
H5	SPR	13	2	2	6	7	13	13
	SUM	50	3	6	12	26	47	50
	AUT	18	3	4	8	11	18	18
	Total	74	3	6	14	31	64	74
