# Supplementary material for: Environmental DNA Metabarcoding Reveals Diversity and Spatiotemporal Variations of Benthic Invertebrates in Hun River Basin, China
Source: Biology (Basel). 2026 Sep 10;15(18):1597. doi: 10.3390/biology15181597 (PMC13604782; doi:10.3390/biology15181597)
Supplement: Supplementary file 1 [file biology-15-01597-s001.zip › Table S2. Checklist of taxonomically annotated taxa for benthic invertebrates in the Hun River basin.pdf]

Table S2. Checklist of taxonomically annotated taxa for benthic invertebrates in the Hun River basin.

Phylum	Class	Order	Family	Genus	Species	Seasons	Sites
Annelida	Hirudinea	Arhynchobdellida	Erpobdellidae	*Erpobdella*	*Erpobdella octoculata*	SPR、SUM	H1、H2
	Oligochaeta	Haplotaxida	Naididae	*Branchiura*	*Branchiura sowerbyi*	SPR	H2
				Nais	*Nais badia*	SUM	H1
					Nais communis	AUT	H4
					Nais simplex	SPR、AUT	H2
				Paranais	*Paranais frici*	SPR	H2
				Spirosperma	*Spirosperma ferox*	SUM、AUT	H1、H3、H4、H5
Arthropoda	Branchiopoda	Anomopoda	Daphniidae	*Simocephalus*	*Simocephalus himalayensis*	SUM	H1、H3、H4、H5
	Insecta	Coleoptera	Dryopidae	*Dryops*	*Dryops ernesti*	SUM	H1、H2、H5
			Dytiscidae	*Hydaticus*	*Hydaticus incertus*	SUM	H1、H5
					Hydaticus rhantoides	SUM	H2、H3
				Hydroporus	*Hydroporus puberulus*	SPR	H3、H4
				Ilybius	*Ilybius fenestratus*	SPR	H2
			Elmidae	*Stenelmis*	*Stenelmis* sp.	SPR	H3、H5
			Hydrophilidae	norank1	*Hydrophilidae* sp.	SPR	H1
			Psephenidae	norank2	*Psephenoides* sp.	SUM	H1
		Diptera	Chironomidae	*Acricotopus*	*Acricotopus longipalpus*	SUM	H1、H2、H4
					Acricotopus sp.	SUM	H1
					Acricotopus zhalingensis	SUM	H1、H2、H3
				Brillia	*Brillia bifida*	SPR	H1、H2
					Brillia flavifrons	SUM	H1、H5
				Cardiocladius	*Cardiocladius fuscus*	SUM	H5
				Chironomus	*Chironomus circumdatus*	SUM、AUT	H1-H5
					Chironomus melanotus	SUM	H1、H3、H4、H5

Phylum	Class	Order	Family	Genus	Species	Seasons	Sites
					Chironomus plumosus	SPR	H5
					Chironomus sp.	AUT	H4
				Corynoneura	*Corynoneura arctica*	SPR、SUM	H2、H3
					Corynoneura edwardsi	SUM	H1
				Cricotopus	*Cricotopus bicinctus*	SUM	H1
					Cricotopus flavozonatus	SUM	H1
					Cricotopus montanus	SUM	H2
					Cricotopus perniger	SUM	H5
					Cricotopus trifascia	AUT	H2、H4、H5
				Cyphomella	*Cyphomella cornea*	SUM	H1、H2、H3
				Diamesa	*Diamesa* sp.1XL	SPR	H5
					Diamesa sp.TP12	SUM	H1、H4、H5
				Dicrotendipes	*Dicrotendipes nervosus*	SPR	H3、H4、H5
					Dicrotendipes pelochloris	AUT	H2
				Doithrix	*Doithrix* sp.TP31	SUM	H3、H4、H5
				Eukiefferiella	*Eukiefferiella brevicalcar*	SUM	H2
				Euryhapsis	*Euryhapsis fuscipropes*	SUM	H2、H3、H4、H5
				Harnischia	*Harnischia fuscimana*	AUT	H2
				Heleniella	*Heleniella nebulosa*	SUM	H4、H5
				Limnophyes	*Limnophyes* sp.T19	SUM、AUT	H1-H5
				Metriocnemus	*Metriocnemus albolineatus*	SUM	H1、H4、H5
				Micropsectra	*Micropsectra* sp.TP2	SUM	H2、H3、H4、H5
				Monodiamesa	*Monodiamesa* sp.	SPR	H2
				Neozavrelia	*Neozavrelia mongolensis*	SPR	H5
				Nilodorum	*Nilodorum tainanus*	AUT	H5

Phylum	Class	Order	Family	Genus	Species	Seasons	Sites
				Orthocladius	*Orthocladius kanii*	SUM	H1
					Orthocladius lignicola	SPR	H2
					Orthocladius nitidoscutellatus	SUM	H1-H5
					Orthocladius rivulorum	SUM	H1、H4
				Parachironomus	*Parachironomus arcuatus*	SPR	H5
				Paracladius	*Paracladius* sp.T20	SUM	H2、H3
				Parakiefferiella	*Parakiefferiella gracillima*	SUM、AUT	H5
				Paratanytarsus	*Paratanytarsus grimmii*	SUM、AUT	H1、H2、H4
					Paratanytarsus inopertus	SPR	H3
				Parorthocladius	*Parorthocladius concretus*	SPR	H2
				Potthastia	*Potthastia gaedii*	SUM	H5
				Polypedilum	*Polypedilum arcuatum*	SUM	H1
					Polypedilum masudai	AUT	H2
					Polypedilum scalaenum	AUT	H5
					Polypedilum vanderplanki	SUM	H1、H4
				Procladius	*Procladius choreus*	SUM	H2
					Procladius crassinervis	SUM	H4、H5
					Procladius rufovittatus	SUM	H2
				Propsilocerus	*Propsilocerus akamusi*	AUT	H1-H5
				Rheocricotopus	*Rheocricotopus atripes*	SUM	H1
					Rheocricotopus effusus	SPR	H4
					Rheocricotopus nemoacrostichalis	SUM	H1
					Rheocricotopus sp.12XL	SUM	H4、H5
					Rheocricotopus taiwanensis	SUM	H1
				Rheopelopia	*Rheopelopia maculipennis*	SUM	H2、H5

Phylum	Class	Order	Family	Genus	Species	Seasons	Sites
				Smittia	*Smittia aterrima*	SUM	H5
				Stenochironomus	*Stenochironomus tobaduodecimus*	AUT	H5
				Stictochironomus	*Stictochironomus akizukii*	SUM	H1-H5
					Stictochironomus juncaii	SUM	H2
				Tanytarsus	*Tanytarsus sinuatus*	SUM	H1
					Tanytarsus takahashii	SPR	H5
				Thienemanniella	*Thienemanniella nipponica*	SUM	H1、H2
					Thienemanniella sp.T12	SUM	H1
				Tvetenia	*Tvetenia* sp.TP21	SUM	H4
			Culicidae	*Aedes*	*Aedes togoi*	SUM	H4、H5
			Dixidae	*Dixella*	*Dixella* sp.	SUM	H1、H2、H4、H5
			Ephydridae	*Parydra*	*Parydra coarctata*	SUM	H1
			Stratiomyidae	*Odontomyia*	*Odontomyia guizhouensis*	SUM	H1
			Tabanidae	*Chrysops*	*Chrysops caecutiens*	SPR	H1
					Chrysops flavocinctus	SUM、AUT	H1、H5
					Chrysops nigripes	SUM	H1、H2、H4、H5
					Chrysops relictus	SUM	H4、H5
				Tabanus	*Tabanus birmanicus*	SUM	H1
		Ephemeroptera	Baetidae	*Procloeon*	*Procloeon pennulatum*	SPR	H3
				Tenuibaetis	*Tenuibaetis* sp.	SUM	H1-H5
			Ephemerellidae	*Drunella*	*Drunella submontana*	AUT	H5
				Ephemerella	*Ephemerella atagosana*	SPR	H5
				Torleya	*Torleya mikhaili*	SPR	H5
			Heptageniidae	*Afronurus*	*Afronurus yixingensis*	SUM	H2
				Epeorus	*Epeorus bispinosus*	SUM	H3

Phylum	Class	Order	Family	Genus	Species	Seasons	Sites
				Parafronurus	*Parafronurus you*	SUM	H1
			Leptophlebiidae	*Choroterpes*	*Choroterpes yixingensis*	SUM	H1
			Siphlonuridae	*Siphlonurus*	*Siphlonurus immanis*	SUM、AUT	H1、H4、H5
			Vietnamellidae	*Vietnamella*	*Vietnamella dabieshanensis*	SUM	H2、H3
		Hemiptera	Belostomatidae	*Diplonychus*	*Diplonychus esakii*	SUM	H4
			Fulgoridae	*Lycorma*	*Lycorma delicatula*	SUM	H1、H5
			Nepidae	*Nepa*	*Nepa hoffmanni*	SUM	H1、H2
		Odonata	Chlorocyphidae	*Indocypha*	*Indocypha catopta*	SUM	H3
			Coenagrionidae	*Coenagrion*	*Coenagrion lunulatum*	SUM、AUT	H4、H5
				Ceriagrion	*Ceriagrion nipponicum*	SUM	H1
			Gomphidae	*Sinictinogomphus*	*Sinictinogomphus clavatus*	SUM	H1
			Libellulidae	*Sympetrum*	*Sympetrum striolatum*	SUM	H1
			Platycnemididae	*Calicnemia*	*Calicnemia sinensis*	SUM	H1
				Copera	*Copera marginipes*	SUM	H2、H4、H5
		Plecoptera	Capniidae	*Capnia*	*Capnia yunnana*	SPR	H5
			Diamphipnoidae	*Diamphipnopsis*	*Diamphipnopsis* sp.	SUM	H1
			Leuctridae	*Perlomyia*	*Perlomyia isobeae*	SUM	H4、H5
			Perlidae	*Flavoperla*	*Flavoperla* sp.	SUM	H4、H5
			Perlodidae	*Perlodidae*	*Arcynopteryx dichroa*	AUT	H1
			Taeniopterygidae	*Taeniopteryx*	*Taeniopteryx auberti*	SUM	H1
					Taeniopteryx nebulosa	SUM	H4
		Trichoptera	Ecnomidae	*Ecnomus*	*Ecnomus gigantius*	SUM	H1、H5
			Glossosomatidae	*Agapetus*	*Agapetus chinensis*	SUM	H1
				Glossosoma	*Glossosoma* sp.	SUM	H1-H5
					Glossosoma sp.2	SUM	H2

Phylum	Class	Order	Family	Genus	Species	Seasons	Sites
			Goeridae	*Goera*	*Goera martynowi*	SUM	H1、H5
			Hydropsychidae	*Cheumatopsyche*	*Cheumatopsyche* sp.CN1	SUM	H1、H5
					Cheumatopsyche sp.CN12	SUM	H4
				Hydropsyche	*Hydropsyche kozhantschikovi*	SUM	H5
				Hydromanicus	*Hydropsyche* sp.CN1	SUM	H1-H5
			Hydroptilidae	*Oxyethira*	*Oxyethira ecornuta*	AUT	H4
			Leptoceridae	*Ceraclea*	*Ceraclea brachyacantha*	SPR	H2
				Leptocerus	*Leptocerus colophallus*	SUM、AUT	H2、H3、H4、H5
				Mystacides	*Mystacides elongata*	SUM、AUT	H4、H5
				Oecetis	*Oecetis minuscula*	AUT	H4
			Limnephiloidea	*Neophylax*	*Neophylax* sp.CN1	SUM	H1
			Polycentropodidae	*Polyplectropus*	*Polyplectropus acutus*	SPR	H1-H5
				Nyctiophylax	*Nyctiophylax gracilis*	SUM	H1
					Nyctiophylax macrorrhinus	SUM	H5
			Rhyacophilidae	*Rhyacophila*	*Rhyacophila haplostephana*	SPR	H1、H2、H5
					Rhyacophila sp.	AUT	H5
					Rhyacophila_triangularis	SUM	H4、H5
					Rhyacophila vascula	SUM	H1
			Psychomyiidae	*Tinodes*	*Tinodes* sp.	SUM	H1、H2、H5
	Malacostraca	Amphipoda	Gammaridae	*Gammarus*	*Gammarus lacustris*	SUM	H1、H3、H4、H5
					Gammarus nipponensis	SUM	H1、H5
					Gammarus qiani	AUT	H5
		Decapoda	Atyidae	*Neocaridina*	*Neocaridina denticulata*	AUT	H5
			Potamidae	*Yarepotamon*	*Yarepotamon gracillipa*	SPR	H2
Mollusca	Bivalvia	Sphaeriida	Sphaeriidae	*Pisidium*	*Pisidium nitidum*	SUM	H1

Phylum	Class	Order	Family	Genus	Species	Seasons	Sites
					Pisidium sp.	SPR	H2
		Unionida	Unionidae	*Unio*	*Unio douglasiae*	SUM	H3
				Anodonta	*Anodonta arcaeformis*	SPR	H2
					Anodonta woodiana	SPR	H2
		Venerida	Cyrenidae	*Corbicula*	*Corbicula fluminea*	SUM	H3、H4、H5
	Gastropoda	Architaenioglossa	Viviparidae	*Sinotaia*	*Sinotaia purificata*	SUM、AUT	H3、H4
		Basommatophora	Lymnaeidae	*Radix*	*Radix plicatula*	SUM、AUT	H1、H5
					Radix Qinghai	SUM	H1
			Planorbidae	*Hippeutis*	*Hippeutis cantori*	SUM	H1
		Sorbeoconcha	Pachychilidae	*Sulcospira*	*Sulcospira paludiformis*	SPR、SUM、AUT	H1-H5
			Pomatiopsidae	*Tricula*	*Tricula montana*	SUM	H5
			Semisulcospiridae	*Hua*	*Hua scrupea*	AUT	H4
